# Dr. Morris N. Bemus

**Published:** 1918-02

**Authors:** 


**Dr. Morris N. Bemus** of Jamestown died Dec. 7, age 55 years, after an illness of ten days. Dr. Bemus was the son of the late Dr. William P. Bemus, a pioneer physician of the county. He made application to join the medical staff of the National Army but was not accepted on account of age, although he passed a perfect physical examination.

